# Supplementary material for: Nominally identical microplastic models differ greatly in their particle-cell interactions
Source: Nat Commun. 2024 Jan 31;15:922. doi: 10.1038/s41467-024-45281-4 (PMC10830523; doi:10.1038/s41467-024-45281-4)
Supplement: Supplementary file 1 — Supplementary Information [file 41467_2024_45281_MOESM1_ESM.pdf]

Supplementary information for

Nominally identical microplastic models differ greatly in their particle-cell interactions

Simon Wieland[†,1,2], Anja FRM Ramsperger[†,1,2], Wolfgang Gross[†,1], Moritz Lehmann[3], Thomas Witzmann[4], Anja Caspari[4], Martin Obst[5], Stephan Gekle[3], Günter K Auernhammer[4], Andreas Fery[4,6], Christian Laforsch[*,2], Holger Kress[*,1]

[1] Biological Physics, University of Bayreuth, Bayreuth, Germany
[2] Animal Ecology I and BayCEER, University of Bayreuth, Bayreuth, Germany
[3] Biofluid Simulation and Modeling – Theoretical Physics VI, University of Bayreuth, Bayreuth, Germany
[4] Leibniz Institut für Polymerforschung Dresden e. V., Institute of Physical Chemistry and Polymer Physics, Dresden, Germany
[5] Experimental Biogeochemistry, BayCEER, University of Bayreuth, Bayreuth, Germany
[6] Physical Chemistry of Polymeric Materials, Technische Universität Dresden, Dresden, Germany
[†] These authors contributed equally to this work.
[*] These authors jointly supervised this work. Corresponding authors: christian.laforsch@uni-bayreuth.de, holger.kress@uni-bayreuth.de

This PDF file includes:

Supplementary Notes 1 and 2
Supplementary Figures 1 to 18
Supplementary Tables 1 to 4
Supplementary References

Supplementary Notes

Supplementary Note 1: Particle SEM micrographs

The size and shape of the microplastic particles were investigated by using scanning electron microscopy (SEM). Most microplastic particle types deviated by less than 3% from the nominal diameter specified by the manufacturers. However, TJ, and PX deviated by 12% and 4% (Table 1). Furthermore, we found that the surface morphologies of the pristine microplastic particles show clear differences between the particle types (Figure 1, Supplementary Table 1). ST and TJ particles had the most irregular surfaces compared to all other particles. We observed indentations on the surfaces of the TJ particles and elevations on the PX and TJ particles. The other particles displayed in Figure 1 (MM-C, MM, MG, KI and TS) were highly spherical with smooth surfaces, although MG seemed to be covered by a net-like structure. The environmentally exposed particles (MM-SW2 and MM-SW4, MM-FW2, and MM-FW4) were coated heterogeneously by biomolecules and organic debris (eco-corona) as shown previously[1] and further analyzed by STXM and XPS measurements (Supplementary Figure 4, Supplementary Tables 2 and 3). The average diameter of MM-SW2, MM-SW4, and MM-FW4 increased slightly by a few nanometers, indicating a thin layer of biomolecules[1]. Furthermore, the standard deviation of the mean diameter of all environmentally exposed microplastic particles substantially increased, indicating that they became less monodisperse. Accordingly, also their eccentricity and surface roughness increased.

Supplementary Note 2: Validation of the flow profile

To validate that the derived velocity profile $v_{\mathrm{x,par}}(y, z)$ given by equation (15) matches the flow field, which is established in the channels, we measured the velocity profile inside the channel with a high-speed camera and particle tracking. A small number of carboxylated polystyrene MPs with a diameter of 1 µm (micromer, micromod Partikeltechnologie GmbH, Rostock, Germany) was dispersed in MilliQ water at a concentration of approximately 2×10^5 µL^{-1}. The solution was pumped through the channel at flow rates between 0.2 µL s^{-1} and 2 µL s^{-1}, which was controlled by setting the motor velocity according to equation (8). The resulting flow field $v_{\mathrm{x,par}}(y, z)$ (equation (15)) was measured on an inverted, motorized microscope (Nikon Eclipse Ti, Nikon, Tokyo, Japan) with a 40x water immersion objective with a high numerical aperture to minimize the focal depth (CFI Apo LWD 40× WI λS, Nikon, NA = 1.15). Brightfield images of the flow at various y and z positions were captured with a high-speed camera (IDT Nx4-S2, Integrated Design Tools, Pasadena, California) at frame rates of up to 2000 Hz.

To detect the particles automatically, the images were filtered with a spatial band pass filter in order to reduce noise and to remove any signals on length scales larger than the particles. Secondly, the images were filtered with a moving median filter in time to single out all stationary objects, e.g. sensor dust. These filtered images were then subtracted from the images. The moving particles were then detected with a custom-written peak-finding routine and tracked with a particle tracking algorithm[2]. The code was implemented in MATLAB 2019b. For every position inside the channel, the velocity of typically 50-100 particles was averaged. Slight deviations from the mean value could be attributed to particles located in slightly lower or higher z positions than the main focal plane, which were still sharp enough to be tracked. The measured flow profile $v_{\mathrm{x}}(y, z)$ was in agreement with the theoretical prediction (Supplementary Figure 11).

As we used different flow rates during the rupture experiments, we also tested whether the velocity inside the channel scales linearly with the flow rate as expected from equations (14) and (15). The

high-speed camera was fast enough to capture particles with velocities of up to 3.5 mm s^{-1} at a flow rate of 2 μL s^{-1} with sufficiently short frame time intervals for the particle tracking algorithm. Up to 2 μL s^{-1}, we found perfectly linear scaling, demonstrating that the pressure even at relatively low flow rates is high enough to properly close the check valves in the tubing system (see Supplementary Figure 12). During the calibration experiments and during the cell experiments, we observed straight particle trajectories far away from the cells, indicating laminar flow in the channels.

Since the measured velocity profile $v_{\mathrm{x}}(y, z)$, both in shape and magnitude, was in excellent agreement with the theoretical prediction (see Supplementary Figures 11 and 12), we concluded that equation (17) can be used to correct for the parabolic height profile.

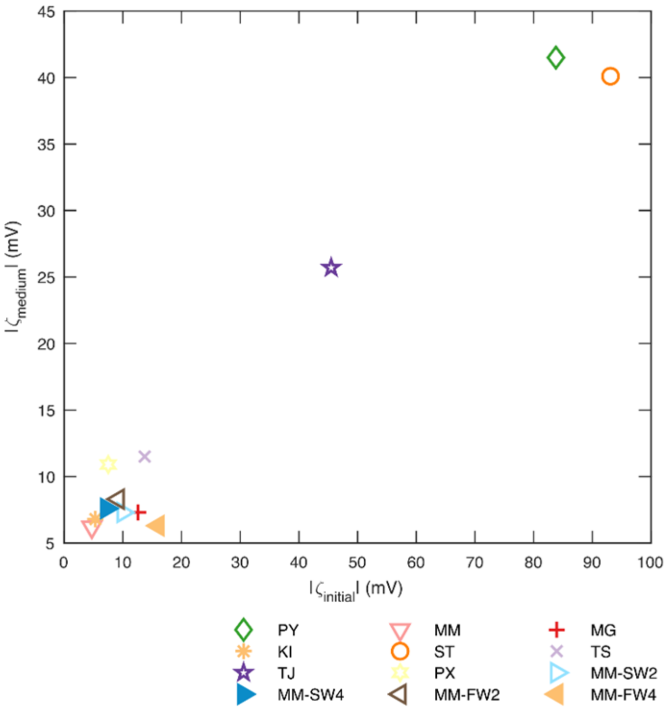

Supplementary Figure 1: ζ-potential before and after incubation in cell culture media.
The ζ-potential of microplastic particles after an incubation of 2 h in cell culture media ζ_{medium} was strongly correlated to their initial ζ-potential ζ_{initial} (Pearsons's $R = 0.8$, $P = 0.004$). Detailed values of the ζ-potential are given in Supplementary Table 1. Source data are provided as a Source Data file.

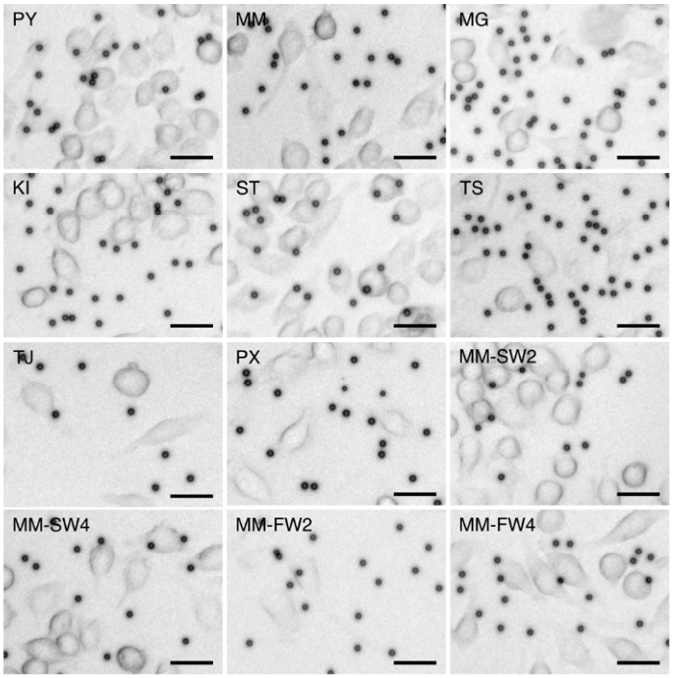

Supplementary Figure 2: Colloidal stability of microplastic particles.
All particle types showed a high colloidal stability during cell experiments, no significant aggregation of beads occurred. Here, example images of particles and cells during the microfluidic experiments are shown. These images were acquired after the sedimentation phase, before the flow was turned on to flush the particles off the cells. Scale bar: 25 µm.

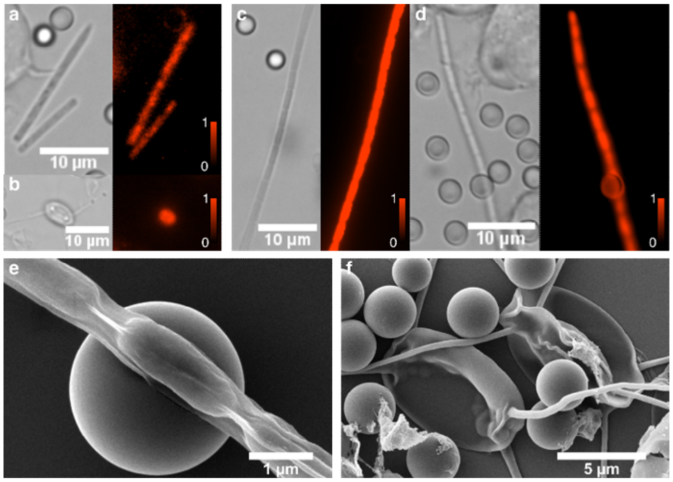

Supplementary Figure 3: Organisms in environmental media.
Various microorganisms were present in the environmental media. (**a**) and (**b**) show examples of freshwater organisms in brightfield and fluorescence microscopy (Texas Red channel, exc. 542-582 nm, em. 604-644 nm; color scales represent fluorescence in arbitrary units). (**c**) and (**d**) show examples of saltwater organisms in brightfield and fluorescence microscopy. (**e**) and (**f**) show scanning electron micrographs of freshwater organisms. (**a**) possibly shows a diatom. (**b**) and (**f**) possibly show green algae of the genus *Lagerheimia*. (**c**), (**d**), and (**e**) possibly show examples of cyanobacteria. The autofluorescence of the microorganisms in the Texas Red channel might be caused by chlorophyll.

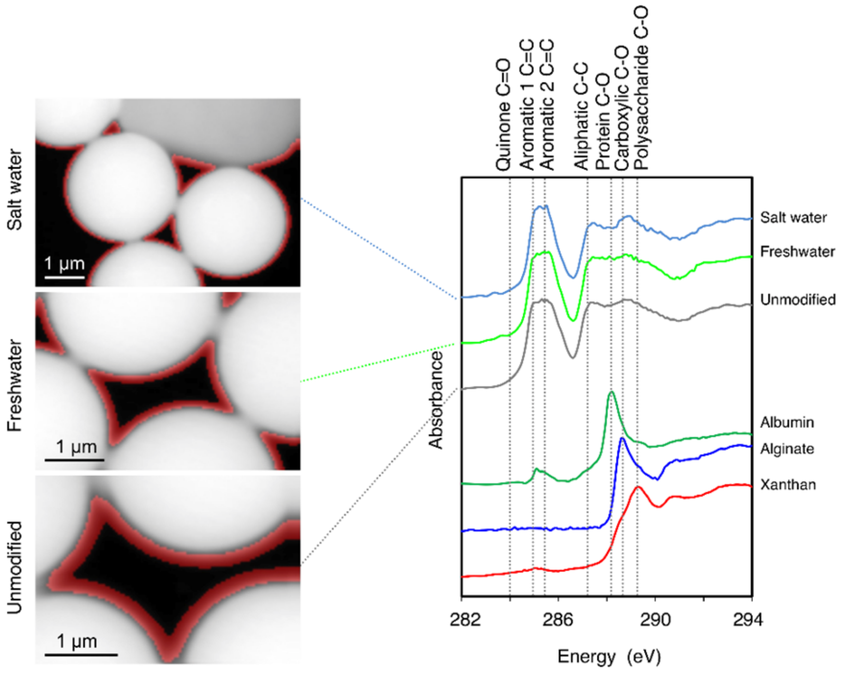

Supplementary Figure 4: STXM analysis of the eco-corona.

C1*s* NEXAFS spectra extracted from Scanning Transmission X-ray Microscopy (STXM) datasets from regions directly at the surface of PS beads that were previously incubated in seawater, freshwater and deionized water respectively. The surface regions, highlighted in red, were selected in the STXM images based on the average optical density (OD) range of 0.1-0.9 across the C1*s* absorption edge, which is equivalent to a cumulative thickness of up to 100 nm, arranged tangentially around the PS beads. These regions are marked in red in the respective STXM images. The equivalent thickness was calculated using the atomic scattering factors[3], the formula C_8H_8 and an assumed density of 1.09 g cm^{-3} of the polystyrene. All 3 spectra were decomposed into a sum of individual gaussian peaks plus the ionization edge modelled as an arctan function. A minimum of 7 analytical peaks was required and used for fitting the respective spectra: 284.0 eV (quinone C=O), 285.0 and 285.4 eV (aromatic C=C), 287.4 eV (aliphatic C-C), 288.2 eV (protein C-O), 288.9 eV (carboxylic C-O), 289.5 (polysaccharide C-O). Peak energies and widths were optimized and fixed at the same values for all 3 spectra, whereas the respective peak areas were fitted (Supplementary Table 2) using the peak fitting algorithm of Athena[4]. For illustrating the peak energies that are subtle only in the spectra of the thin regions of the eco-corona around the PS bead, 3 reference materials are presented as well, for protein (albumin), acidic polysaccharides (alginate) and neutral polysaccharides (xanthan)[5]. The dashed grey lines indicate the respective peak energies of the gaussians used for fitting. The data lines of the spectra were offset for clarity.

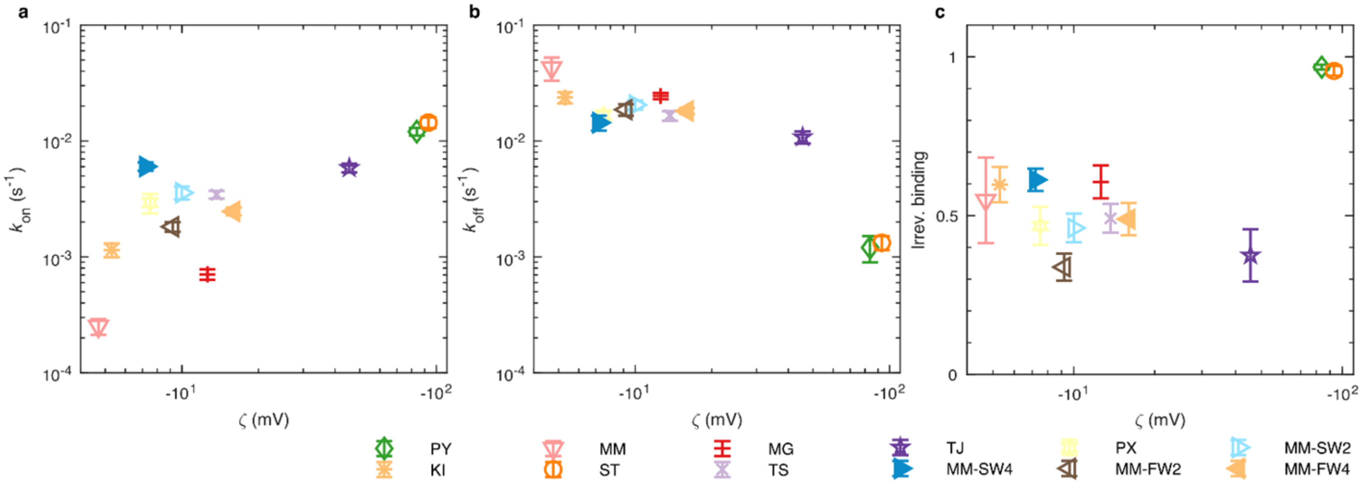

Supplementary Figure 5: Binding kinetics of microplastic particles to coverslips.
Binding kinetics to coverslips significantly differed between samples (k_{on}: Kruskal-Wallis test, two-sided P = 8.69 × 10^{-16}; k_{off}: Kruskal-Wallis test, two-sided P = 5.77 × 10^{-10}; Irreversible binding: Kruskal-Wallis test, two-sided P = 2.83 × 10^{-6}). We measured rates of a similar magnitude as for the binding of microplastic particles to cells. This means that particles which strongly bound to cells also bound strongly to coverslips. However, particle-coverslip adhesion was in general slightly weaker than particle-cell adhesion (compare Figure 2). This was reflected by a generally lower k_{on} (**a**) to coverslips, a higher k_{off} (**b**) from coverslips, and a generally lower fraction of irreversible binding events (**c**). Like for the binding to cells, the binding kinetics were strongly correlated to the particles' ζ-potential. With increasing negative ζ-potential k_{on} increased (Pearson's R = 0.9, two-sided P = 9.4 × 10^{-6}), k_{off} decreased (Pearson's R = -0.8, two-sided P = 0.003), and the fraction of irreversible binding events increased (Pearson's R = 0.8, two-sided P = 0.003). In all panels, error bars represent standard error of mean of n = 9 measurements (for each measurement, on average 550 particles were analyzed). Source data are provided as a Source Data file.

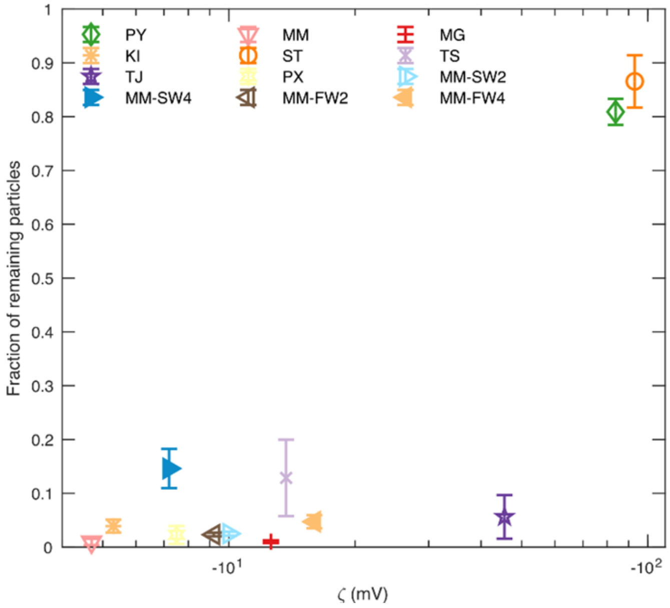

Supplementary Figure 6: Adhesion strength of microplastic particles to coverslips. Adhesion of particles to coverslips significantly differed between samples (Kruskal-Wallis test: two-sided $P = 1.24 \times 10^{-10}$). Particles that strongly adhered to cells also strongly adhered to coverslips and vice versa. Generally, the fraction of microplastic particles remaining on coverslips after flushing with a hydrodynamic force of 50 pN for 30 s was slightly lower compared to the fraction of particles remaining on cells (compare Figure 3). The fraction of particles remaining on coverslips was correlated to the particles' ζ-potential. With increasing negative ζ-potential, the fraction of remaining particles strongly increased (Pearson's $R = 0.9$, two-sided $P = 9.4 \times 10^{-6}$). Error bars represent standard error of mean of $n = 9$ measurements (for each measurement, on average 550 particles were analyzed). Source data are provided as a Source Data file.

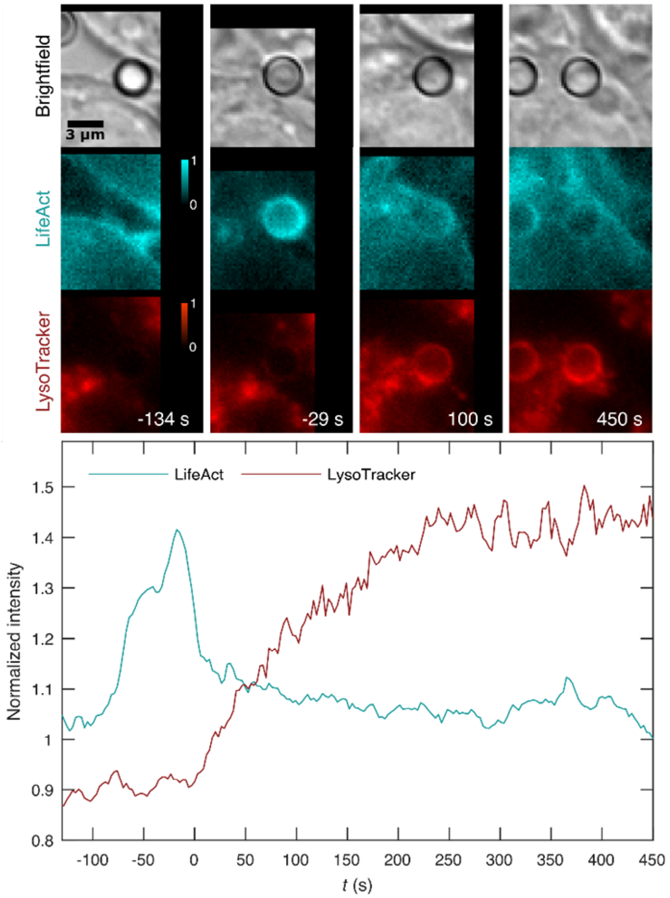

Supplementary Figure 7: Internalization and maturation of microplastic particles.
To test, if the microplastic particles were internalized by phagocytosis or macropinocytosis, we monitored the actin cytoskeleton during particle internalization by cells that were stably transfected with a LifeAct-GFP construct. Since this construct binds to filamentous actin, it is enriched at sites of actin polymerization. Furthermore, we treated the cells with LysoTracker dye, which is enriched in acidic organelles like lysosomes. In this example, the internalization and subsequent maturation of a TS particle is shown. Color scales in the fluorescence images represent fluorescence in arbitrary units. First, the particle interacted with the surface of a cell (t = -134 s). Then we observed a significant peak in the LifeAct intensity around the particle, indicating polymerization of actin filaments (t = -29 s). After the LifeAct signal decayed (t = 0 s), the LysoTracker intensity increased gradually (t = 100 s) until it reached a plateau at the end of the measurement (t = 450 s), showing interactions with lysosomes and the acidification of the internalized particle. Source data are provided as a Source Data file.

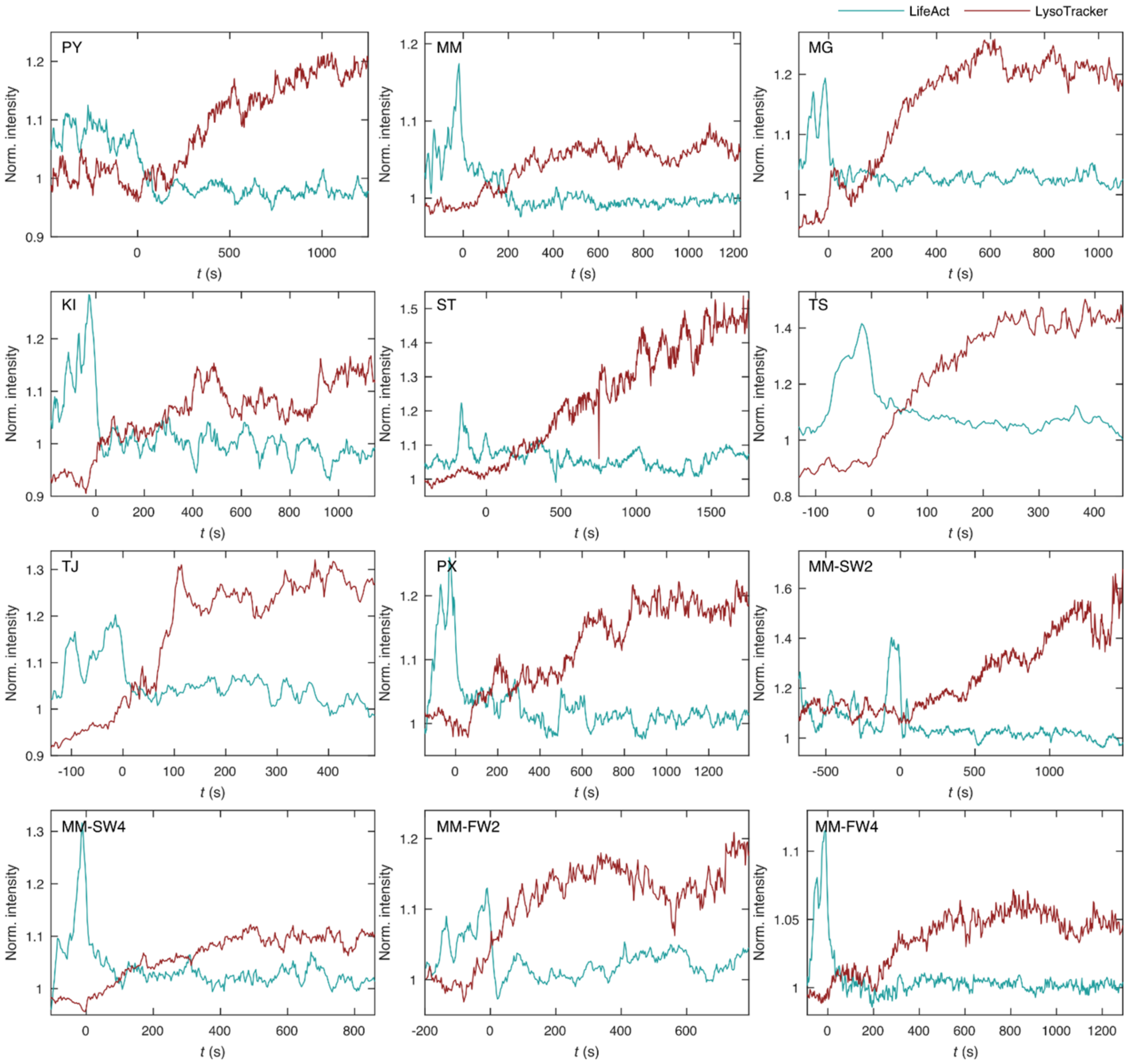

Supplementary Figure 8: Internalization and maturation of all particle types.
All particle types underwent a similar form of internalization and maturation: After the particles interacted with the cells, there was a substantial increase in the LifeAct signal at the site of the particles, indicating that filamentous actin was polymerized. This shows that particles were internalized by an actin-dependent pathway, such as phagocytosis or macropinocytosis. After the LifeAct signal around the particle decayed, indicating depolymerization of the actin filaments, the LysoTracker signal gradually increased over time. This shows that internalized particles interacted with lysosomes and became acidic, undergoing a maturation process that is typical for phagosomes or macropinosomes. Source data are provided as a Source Data file.

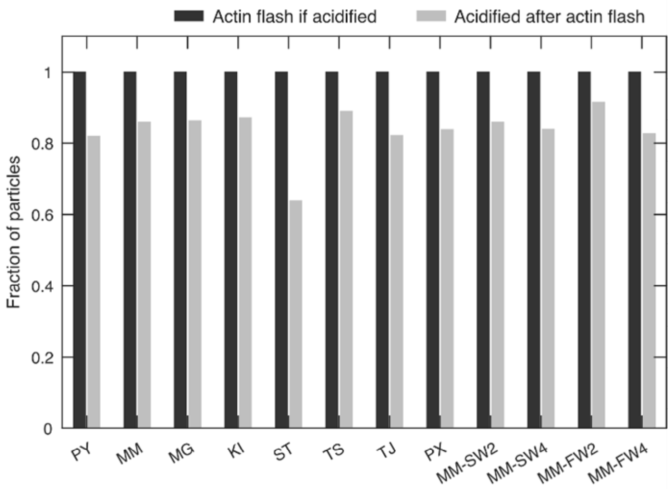

Supplementary Figure 9: Internalization mechanisms of microplastic particles.
All particles that were acidified over the course of a measurement showed an actin peak before the acidification process started, indicating internalization via an actin-dependent pathway (dark bars). This was observed for all particle types. Moreover, most of the particles which at some point during the measurement time showed a LifeAct peak were acidified later (light bars). In cases where particles showed no acidification after a LifeAct peak, the actin-dependent internalization process might not have been completed or the maturation process might not yet have started before the end of a measurement. Overall, these results indicate that the microplastic particles were always internalized via the actin-dependent processes of phagocytosis or macropinocytosis. Number *n* of evaluated uptake processes: PY: 33, MM: 35, MG: 36, KI: 31, ST: 72, TS: 36, TJ: 39, PX: 49, MM-SW2: 35, MM-SW4: 37, MM-FW2: 35, MM-FW4: 23. Source data are provided as a Source Data file.

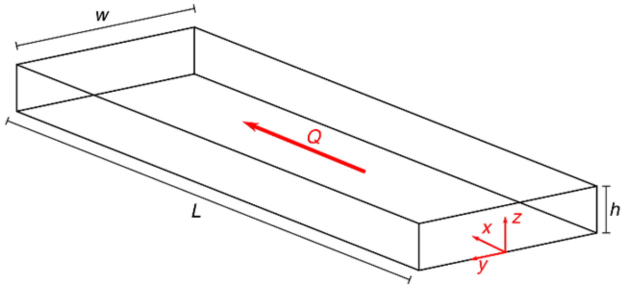

Supplementary Figure 10: Flow geometry in a rectangular channel
Visualization of the channel parameters used to derive the Poiseuille flow profile.

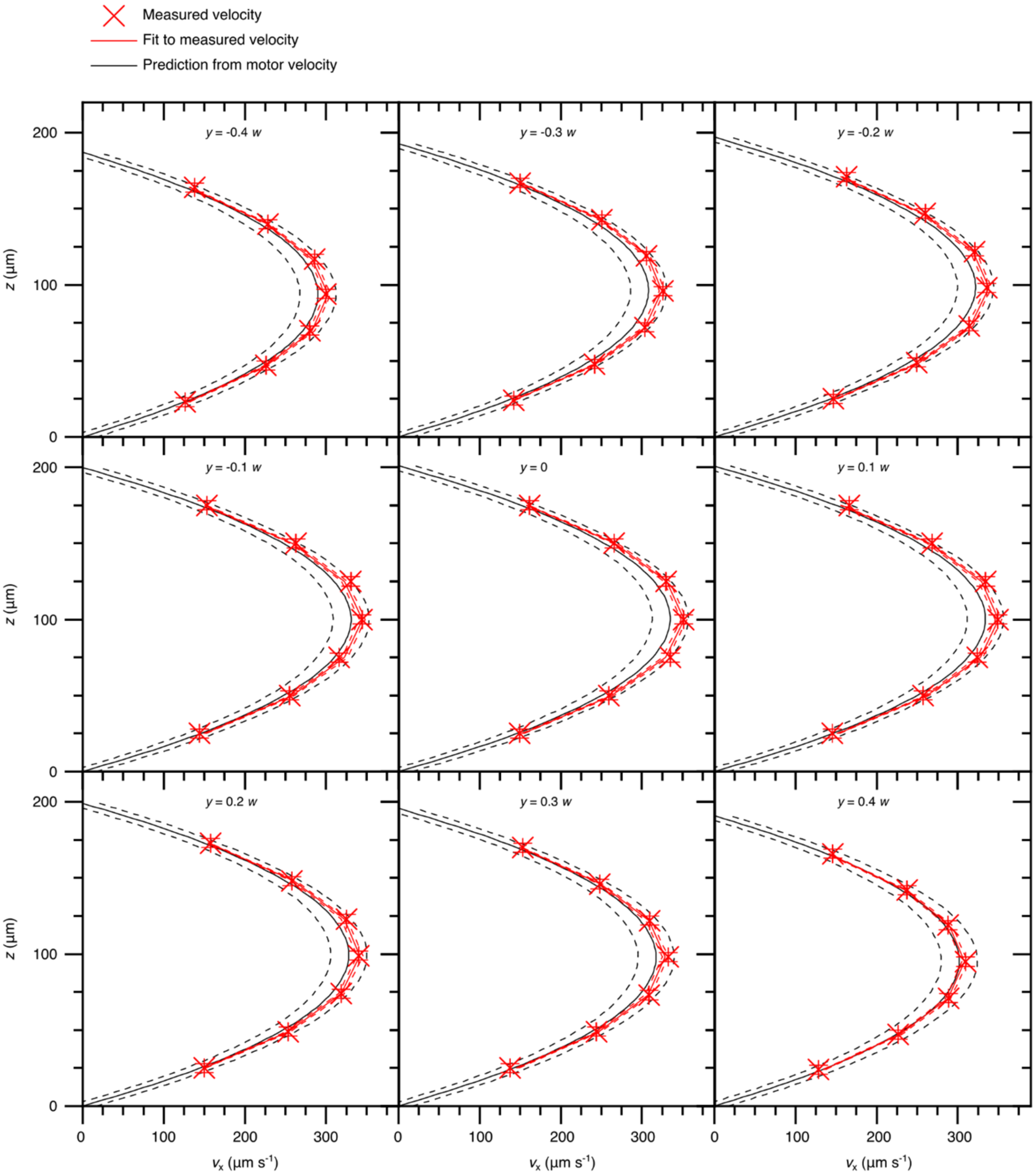

Supplementary Figure 11: Velocity field $v_x(y, z)$ inside a flow channel
Velocity field $v_x(y, z)$ inside a flow channel measured by tracking tracer particles at a flow rate of 0.2 µL s^{-1}. The data points represent mean velocities of typically 50-100 particles. Error bars represent the standard deviation of all measured velocities. Note that the channel was approximately 15 µm higher in the center at $y = 0$ than near the side walls at $y = \pm 0.4\,w$. The

velocity field given by equation (15) was fitted to the measured data with the flow rate in the center as the only free parameter. The measured velocity field matched the velocity field $v_{(\mathrm{x,par})}$ calculated from the motor velocity and the channel geometry within the margin of error. The measured flow field inside the channel matched the flow field predicted from equations (8), (14), and (15) within the margin of error, which was calculated by gaussian error propagation. Notably, this was also the case near the side walls of the channel, where the channel was approximately 15 μm thinner. Source data are provided as a Source Data file.

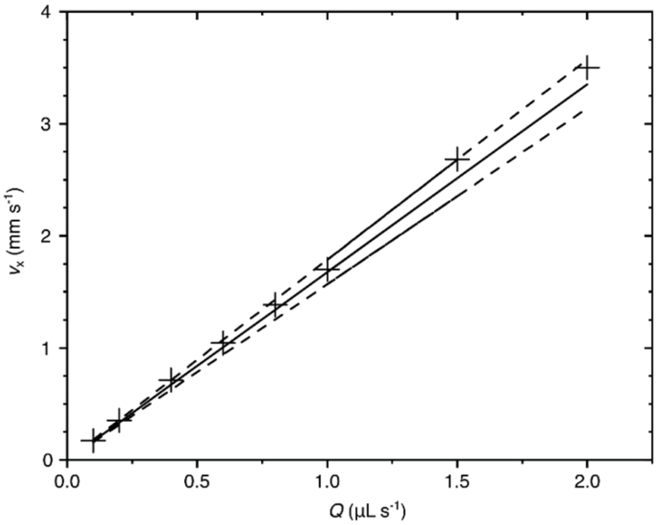

Supplementary Figure 12: Velocity $v_x(0, h/2)$ as a function of the flow rate Q

Velocity $v_x(0, h/2)$ in the middle of the channel as a function of the flow rate Q. The solid line and the dashed lines show the expected flow velocity at the set motor velocity v_m in the middle of the cannel calculated from equations (8), (14), and (15). The error was calculated using Gaussian error propagation. Source data are provided as a Source Data file.

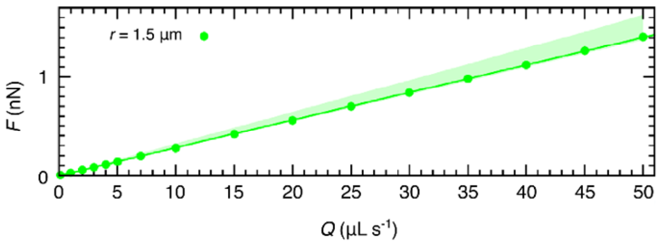

Supplementary Figure 13: The force $F(Q)$ as a function of the flow rate Q.
The filled dots represent the interpolated simulations (case C). The lightly colored range is the corridor between case A and case B. We performed a linear fit (equation (7) with $C_1 = 1$), illustrated as a continuous line.

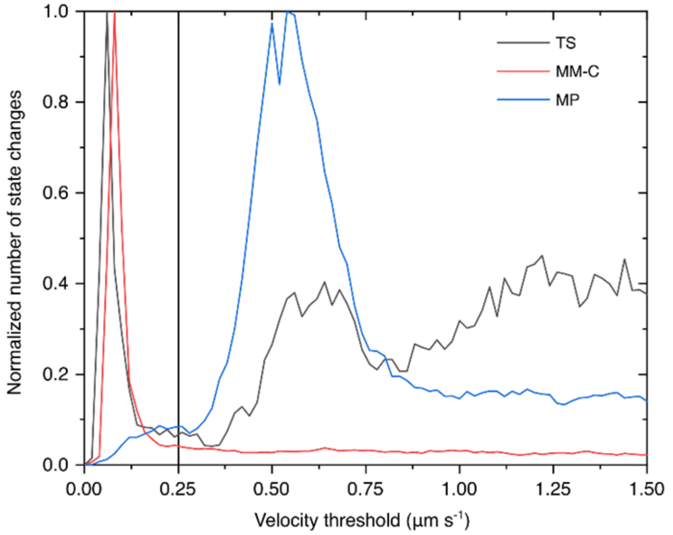

Supplementary Figure 14: Estimation of the threshold velocity.

Number of particle state changes between bound and unbound state as a function of the velocity threshold for exemplary particle types (TS, MM-C and MG). Non-adhesive particles such as MG diffuse in the sample and have velocities which were typically higher than 0.25 µm s^{-1} while the observed velocity of sticky particles such as MM-C was determined by noise and below 0.25 µm s^{-1}. TS particles of which some were bound, and some were unbound showed the same two different regimes in their velocity distribution. Both regimes could be separated by a threshold at 0.25 µm s^{-1}. Source data are provided as a Source Data file.

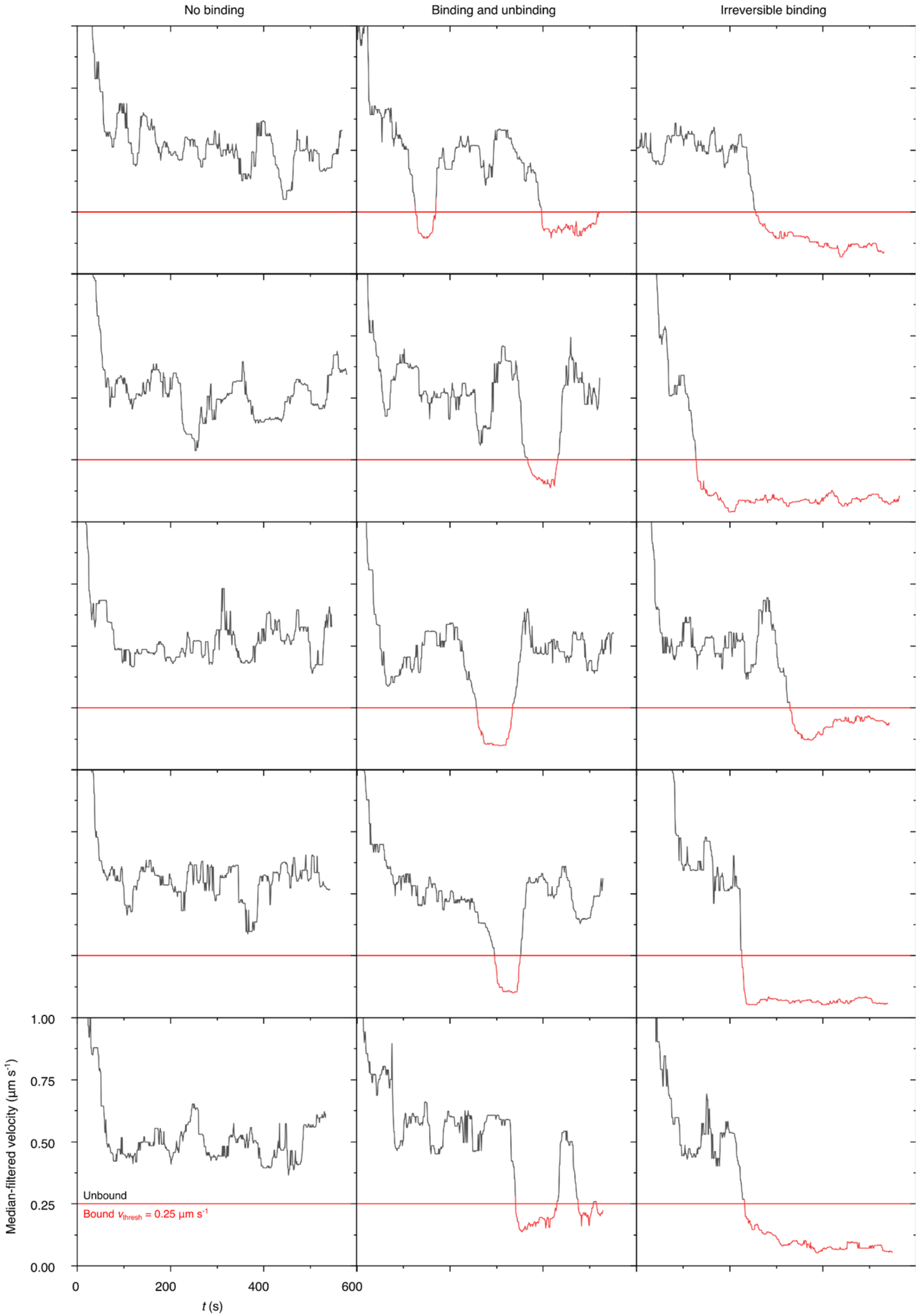
No binding
Binding and unbinding
Irreversible binding
Median-filtered velocity (μm s⁻¹)
1.00
0.75
0.50
0.25
0.00
0
200
400
600
t (s)
Unbound
Bound v_thresh = 0.25 μm s⁻¹

Supplementary Figure 15: Examples of particle trajectories and their binding states. Median filtered particle velocities and their respective binding states. The velocity was filtered with a median filter with a length of 31 s to remove noise. The binding state was determined via thresholding (Supplementary Figure 14) and is color coded in the panels (red: bound ($v < v_{\mathrm{thresh}}$); black: unbound ($v > v_{\mathrm{thresh}}$). The state changes of the particles differed drastically. Some particles never bound (left column), others bound and unbound shortly after the binding event (center column) and some bound and did not detach until the end of the experiment (right column). Source data are provided as a Source Data file.

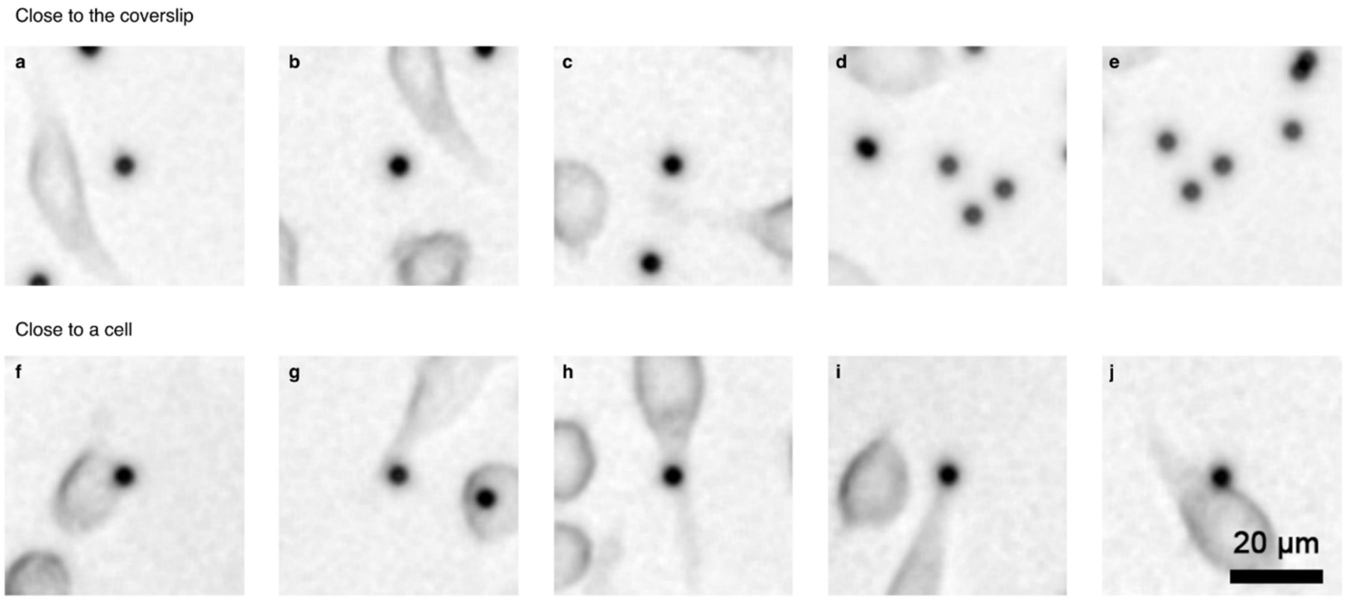

Supplementary Figure 16: Example images used for training the convolutional neural net. The network was trained to classify whether the particle in the center of the image was close to the coverslip (**a-e**) or close to a cell (**f-j**).

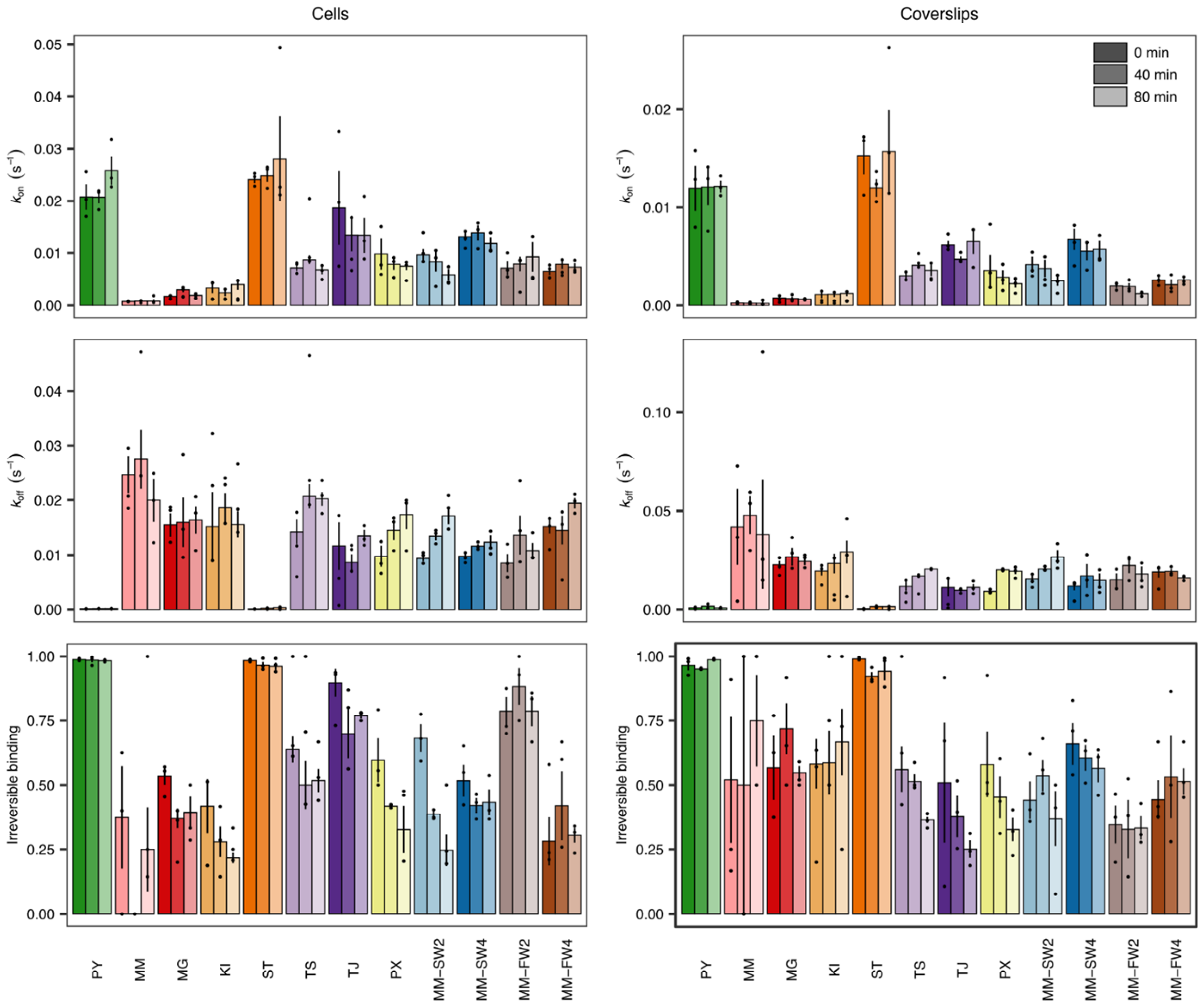

Supplementary Figure 17: Time dependence of binding kinetics.
We performed measurements after $t = 0$ min (dark colors), 40 min (medium colors), and 80 min (light colors) after the particles were added to the microfluidic system to investigate possible interactions of the particles with the image medium. k_{on}, k_{off}, and the fraction of irreversible binding events did not depend on t in most cases. Only the fraction of irreversible binding events to cells decreased slightly with time for PX and MM-SW2 particles. Error bars represent standard error of mean of $n = 3$ individual measurements (indicated as black dots, for each measurement, on average 550 particles were analyzed). Source data are provided as a Source Data file.

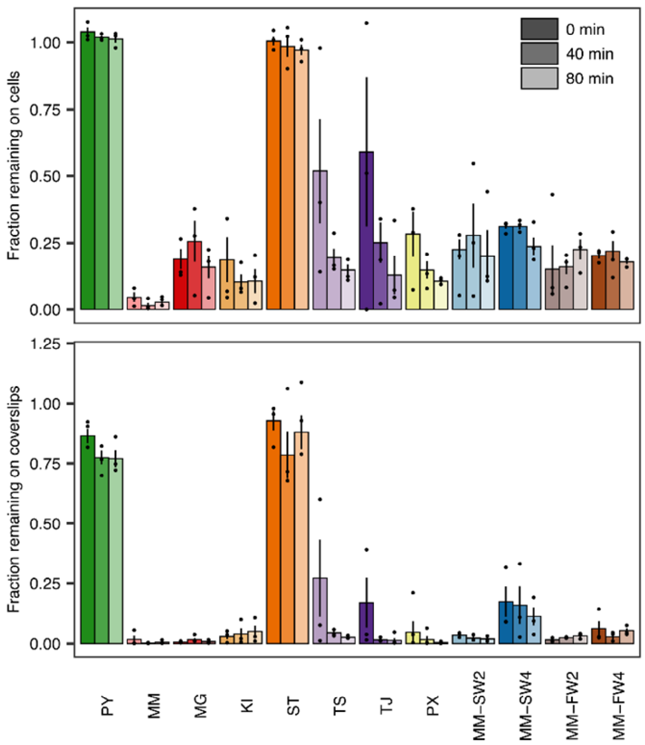

Supplementary Figure 18: Time dependence of adhesion strength.
We performed measurements after $t = 0$ min (dark colors), 40 min (medium colors), and 80 min (light colors) after the particles were added to the microfluidic system to investigate possible interactions of the particles with the image medium. Although some microplastic particles showed a decrease of the average number of remaining particles over time (e.g., TS, TJ), this was not statistically significant. This may indicate that factors other than protein corona formation dominate the variance of the measured adhesive strength. Error bars represent standard error of mean of $n = 3$ individual measurements (indicated as black dots, for each measurement, on average 550 particles were analyzed). Source data are provided as a Source Data file.

**Particle type**	Nominal density (g cm^{-3})	Equivalent diameter (μm)	Eccentricity	Roughness	$\zeta_{initial}$ (mV)	ζ_{medium} (mV)
PY	n.a.	3.08 ± 0.20	1.003 ± 0.001	1.04 ± 0.01	-83.8 ± 0.3	-41.5 ± 0.5
MM	1.03	2.94 ± 0.02	1.005 ± 0.001	1.04 ± 0.01	-4.7 ± 0.3	-6.2 ± 1.0
MG	1.05	2.97 ± 0.11	1.01 ± 0.03	1.05 ± 0.02	-12.6 ± 0.3	-7.3 ± 0.2
KI	n.a.	2.96 ± 0.03	1.005 ± 0.001	1.05 ± 0.01	-5.3 ± 0.5	-6.8 ± 0.4
ST	1.05	3.47 ± 0.26	1.005 ± 0.002	1.06 ± 0.04	-93.1 ± 1.1	-40.1 ± 1.0
TS	1.05	2.84 ± 0.03	1.005 ± 0.004	1.05 ± 0.02	-13.7 ± 0.2	-11.5 ± 0.4
TJ	n.a.	3.36 ± 0.07	1.008 ± 0.004	1.07 ± 0.02	-45.5 ± 0.7	-25.7 ± 6.3
PX	1.06	3.13 ± 0.33	1.02 ± 0.03	1.037 ± 0.004	-7.5 ± 0.4	-10.9 ± 0.5
MM-SW2	-	2.97 ± 0.17	1.04 ± 0.08	1.18 ± 0.15	-10.0 ± 0.7	-7.3 ± 0.4
MM-SW4	-	3.03 ± 0.14	1.02 ± 0.01	1.07 ± 0.04	-7.2 ± 1.3	-7.6 ± 1.3
MM-FW2	-	2.88 ± 0.28	1.005 ± 0.006	1.07 ± 0.04	-9.2 ± 0.5	-8.3 ± 0.5
MM-FW4	-	2.96 ± 0.04	1.01 ± 0.01	1.08 ± 0.07	-16.0 ± 2.3	-6.3 ± 0.7

Supplementary Table 1: Physicochemical particle properties.
The nominal density of the particles was obtained from the respective data sheets. The equivalent diameter, eccentricity, and roughness of the particles were quantified using SEM images. Eccentricity values of 1 correspond to perfect spheres, larger values indicate a more aspherical shape of the particles. Roughness values of 1 correspond to perfectly smooth spheres, larger values indicate more irregular surfaces. Only surface irregularities on length scales larger than 10 nm were detected. For each sample, n = 10 individual particles were analyzed. The ζ-potential of microplastic particles was measured before and after incubation in cell culture medium using a zetasizer. The values of the ζ-potential of microplastic particles after an incubation in cell culture media for 2 h ζ_{medium} are correlated to their initial ζ-potential $\zeta_{initial}$ (Supplementary Figure 2). The ζ-potential measurements were replicated n = 3 times. Errors represent standard deviation. Source data are provided as a Source Data file.

Absorption peak	MM	MM-SW2	MM-FW2
Quinone C=O	0.14	0.05	0.07
1) Aromatic C=C	0.08	0.10	0.08
2) Aromatic C=C	1.17	1.14	1.13
Aliphatic C-C	0.25	0.22	0.25
Protein C-O	0.10	0.07	0.14
Carboxylic COOH	0.18	0.21	0.18
Sugar C-OH	0.07	0.07	0.10

Supplementary Table 2: STXM data.
Fitted peak areas (arbitrary units) from the synchrotron-based scanning transmission X-ray microscopy (STXM). In the absorption spectra we observed signatures from aromatic C=C bonds of polystyrene. The aromatic C=C bonds at two different energies represent the two different types of C-atoms in the aromatic ring of the polymer. Their signatures did not significantly change upon exposure to salt or freshwater. Furthermore, we observed small amounts of quinone C=O bonds that decreased upon exposure to salt or freshwater. The aliphatic C-C bonds were likely part of the bulk polymer and did not change upon exposure to environmental media. Carboxylic groups (COOH) were present on the particles' surface, likely because of oxidation processes. The amount of COOH weakly increased for MM particles exposed to salt water but did not change for freshwater-exposed particles. We observed small amounts of protein-associated C-O bonds and sugar-associated C-OH groups on the MM particles. The amount of these groups significantly increased for MM particles exposed to freshwater, indicating the formation of an eco-corona. However, we could not observe a similar build-up of proteins and sugars on MM particles exposed to salt water. A potential explanation could be that parts of the eco-corona were washed off due to the change in ionic strength in the seawater-incubated sample during the rinsing procedure that was required to avoid salt precipitation during drying of the samples.
Due to the OD range of 0.1-0.9 that was required to obtain a decent spectral quality for peak fitting (which was equivalent to a cumulative thickness of 100 nm tangentially around the particles), the 3 spectra represent a mixture of the polymer itself and a potential eco-corona that is dominated by the polymer. Therefore, we additionally performed X-ray photoelectron spectroscopy, which is more suitable for the analysis of thin surface layers because of its much higher surface sensitivity. (Supplementary Table 3). Source data are provided as a Source Data file.

Element ratio	MM	MM-SW2	MM-FW2
[N]:[C]	-	0.4%	0.3%
[O]:[C]	31.4%	27.6%	30.9%
[Si]:[C]	0.1%	0.7%	0.9%
[Na]:[C]	-	0.2%	-
[Mg]:[C]	-	1.6%	-
[S]:[C]	-	0.2%	-
[Cl]:[C]	-	1.8%	-

Supplementary Table 3: XPS data.
Element concentration ratios were calculated from the energy dispersive XPS spectra. Depending on the element, the detection limit was approximately 0.1%, signals below the detection limit were indicated with “-“. We observed that the surface of environmentally exposed microplastic particles was significantly altered compared to the pristine particles. We observed small amounts of organic nitrogen on MM particles that were incubated in salt and freshwater, which was not present on the pristine MM particles. This could potentially indicate the presence of biomolecules or other natural organic matter, like humid acids, on the microplastics’ surface. Furthermore, there was a substantial increase in the amount of silicon on the environmentally exposed particles, which might be caused by the presence of silicic acid. Silicic acid plays a role in the metabolism of algae like diatoms, but also of other plants and animals. However, since the substrate for the XPS measurements was made from thermally oxidized silicon, this finding should be interpreted with caution. Additionally, we observed a significant decrease of the amount of organically bonded oxygen on the particles surface from the high-resolution C1*s* spectra. This might be related to the production of the MM particles, where possibly non-ionic surfactants, e.g. oligoethoxides, were applied to control the particle growth, which might have been washed out in the environmental media. Furthermore, for the MM particles incubated in salt water, we observed the presence of salts: We could identify significant amounts of sodium, magnesium, sulfur, and chlorine. Source data are provided as a Source Data file.

Particle	PY	MM	MG	KI	ST	TS	TJ	PX	MM-FW2	MM-FW4	MM-SW2	MM-SW4
$k_{(on,cells)}$	A	B	C	BC	A	DE	ADE	DE	DE	D	E	E
$k_{(on,coverslips)}$	A	ABC	B	ABC	A	BC	BC	BC	BC	B	C	BC
$k_{(off,cells)}$	A	B	B	B	A	B	B	B	B	B	B	B
$k_{(off,coverslips)}$	A	ABC	CD	ABCD	A	BCD	D	BCD	BC	BD	BCD	BC
**Irreversible binding events to cells**	A	ABCDE	BC	B	AD	CDE	E	BC	BC	BC	DE	BC
**Irreversible binding events to coverslips**	A	ABC	B	ABC	A	BC	BC	BC	BC	B	C	BC
**Relative attachment to cells**	A	B	BCD	BD	A	BCD	BCD	BCD	BCD	D	BCD	C
**Relative attachment to coverslips**	A	B	BCD	BC	A	BCD	BCD	BCD	BCD	C	BCD	D

Supplementary Table 4: Summary of the statistical analysis.
Statistical testing of the relative attachment between particles and cells as well as between particles and coverslips. Particles of different manufacturers attach significantly different to cells as well as coverslips. The data was tested for a normal distribution using a Shapiro-Wilk test and for a homogeneity of the variances with a Levene test. Differences between particles were identified with a Kruskal-Wallis test together with a Games-Howell post-hoc test. Different letters denote groups of particle types between which significant differences with a two-sided $P < 0.05$ were detected. For example, k_{on} to cells of PY was statistically different to MM, MG, KI, TS, PX, MM-FW2, MM-FW4, MM-SW2, and MM-SW4, but no statistically significant differences existed between PY and ST and TJ. A detailed summary of all statistics is presented in Supplementary Data 1.

Supplementary References

1. Ramsperger, A. F. R. M. *et al.* Environmental exposure enhances the internalization of microplastic particles into cells. *Sci. Adv.* **6**, eabd1211 (2020).
2. Crocker, J. C. & Grier, D. G. Methods of digital video microscopy for colloidal studies. *J. Colloid Interface Sci.* **179**, 298–310 (1996).
3. Henke, B. L., Gullikson, E. M. & Davis, J. C. X-Ray Interactions: Photoabsorption, Scattering, Transmission, and Reflection at E = 50-30,000 eV, Z = 1-92. *At. Data Nucl. Data Tables* **54**, 181–342 (1993).
4. Ravel, B. & Newville, M. ATHENA , ARTEMIS , HEPHAESTUS : data analysis for X-ray absorption spectroscopy using IFEFFIT. *J. Synchrotron Radiat.* **12**, 537–541 (2005).
5. Dynes, J. J. *et al.* Quantitative mapping of chlorhexidine in natural river biofilms. *Sci. Total Environ.* **369**, 369–383 (2006).
