# Supplementary material for: Nominally identical microplastic models differ greatly in their particle-cell interactions
Source: Nat Commun. 2024 Jan 31;15:922. doi: 10.1038/s41467-024-45281-4 (PMC10830523; doi:10.1038/s41467-024-45281-4)
Supplement: Supplementary file 3 — Description of Additional Supplementary Files [file 41467_2024_45281_MOESM3_ESM.pdf]

Description of Additional Supplementary Files

File Name: Supplementary Data 1

Description: **Statistical analysis.** A detailed summary of the statistics presented in this study is provided. All statistical tests were performed using R studio software (version 4.0.2, 2020-06-22) with the packages: "car", “carData”, “rstatix”, “multcompView”). All data were tested for normal distribution (Shapiro-Wilk test) and homogeneity of variances (Levene test). If the Shapiro-Wilk test or the Levene test were significant, a two-sided Kruskal-Wallis test with a Games Howell post hoc test was conducted to check for differences between microplastic particle types. Here, the complete results of the Kruskal-Wallis and Games-Howell post-hoc tests are shown, including the estimate and 95% confidence interval of the population difference and the exact adjusted *P* value for each pair of groups.
