# Supplementary material for: Nominally identical microplastic models differ greatly in their particle-cell interactions
Source: Nat Commun. 2024 Jan 31;15:922. doi: 10.1038/s41467-024-45281-4 (PMC10830523; doi:10.1038/s41467-024-45281-4)
Supplement: Supplementary file 5 — Reporting Summary [file 41467_2024_45281_MOESM5_ESM.pdf]

nature portfolio

Corresponding author(s): Christian Laforsch
Holger Kress

Last updated by author(s): Jan 16, 2024

Reporting Summary

Nature Portfolio wishes to improve the reproducibility of the work that we publish. This form provides structure for consistency and transparency in reporting. For further information on Nature Portfolio policies, see our Editorial Policies and the Editorial Policy Checklist.

Statistics

For all statistical analyses, confirm that the following items are present in the figure legend, table legend, main text, or Methods section.

n/a	Confirmed	
☐	☒	The exact sample size (*n*) for each experimental group/condition, given as a discrete number and unit of measurement
☐	☒	A statement on whether measurements were taken from distinct samples or whether the same sample was measured repeatedly
☐	☒	The statistical test(s) used AND whether they are one- or two-sided<br>*Only common tests should be described solely by name; describe more complex techniques in the Methods section.*
☐	☒	A description of all covariates tested
☐	☒	A description of any assumptions or corrections, such as tests of normality and adjustment for multiple comparisons
☐	☒	A full description of the statistical parameters including central tendency (e.g. means) or other basic estimates (e.g. regression coefficient) AND variation (e.g. standard deviation) or associated estimates of uncertainty (e.g. confidence intervals)
☐	☒	For null hypothesis testing, the test statistic (e.g. *F*, *t*, *r*) with confidence intervals, effect sizes, degrees of freedom and *P* value noted<br>*Give P values as exact values whenever suitable.*
☒	☐	For Bayesian analysis, information on the choice of priors and Markov chain Monte Carlo settings
☒	☐	For hierarchical and complex designs, identification of the appropriate level for tests and full reporting of outcomes
☐	☒	Estimates of effect sizes (e.g. Cohen's *d*, Pearson's *r*), indicating how they were calculated

Our web collection on statistics for biologists contains articles on many of the points above.

Software and code

Policy information about availability of computer code

Data collection	To control the custom-built microfluidic setup, Matlab R2020a was used. All custom code is extensively described in the Methods section and in the Supplementary Information file.
Data analysis	To evaluate the microfluidic experiments, Matlab R2020a was used. All custom code is extensively described in the Methods section and in the Supplementary Information file. Furthermore, microscopy images were evaluated using Fiji ImageJ 1.52i. Statistical analysis was performed using R studio software (version 4.0.2, 2020-06-22).

For manuscripts utilizing custom algorithms or software that are central to the research but not yet described in published literature, software must be made available to editors and reviewers. We strongly encourage code deposition in a community repository (e.g. GitHub). See the Nature Portfolio guidelines for submitting code & software for further information.

Data

Policy information about availability of data

All manuscripts must include a data availability statement. This statement should provide the following information, where applicable:

- Accession codes, unique identifiers, or web links for publicly available datasets
- A description of any restrictions on data availability
- For clinical datasets or third party data, please ensure that the statement adheres to our policy

Data, code, and materials that support the findings of this study are deposited in the Zenodo repository at https://zenodo.org/doi/10.5281/zenodo.10514299. A minimum data set containing the data necessary to reproduce all Figures and Supplementary Figures is provided in the Source Data file.

Research involving human participants, their data, or biological material

Policy information about studies with human participants or human data. See also policy information about sex, gender (identity/presentation), and sexual orientation and race, ethnicity and racism.

Reporting on sex and gender	Not applicable
Reporting on race, ethnicity, or other socially relevant groupings	Not applicable
Population characteristics	Not applicable
Recruitment	Not applicable
Ethics oversight	Not applicable

Note that full information on the approval of the study protocol must also be provided in the manuscript.

Field-specific reporting

Please select the one below that is the best fit for your research. If you are not sure, read the appropriate sections before making your selection.

☒ Life sciences ☐ Behavioural & social sciences ☐ Ecological, evolutionary & environmental sciences

For a reference copy of the document with all sections, see nature.com/documents/nr-reporting-summary-flat.pdf

Life sciences study design

All studies must disclose on these points even when the disclosure is negative.

Sample size	No sample size calculations were performed prior to the experiments. Sample sizes are provided in the Materials and Methods sections, if applicable. Sample sizes were chosen to support meaningful conclusions.
Data exclusions	No data were excluded from the analysis.
Replication	To ensure reproducibility, all experiments determining the binding kinetics and adhesion strength of microplastic particles were replicated three times. For each replicate, three independent measurements were performed, yielding a total of 9 measurements per treatment. For each measurement, on average 550 particles were analyzed. The experiments determining the uptake probability of microplastic particles were replicated three times. For each replicate, the coverslip was screened for 100 particle-cell-interactions (PCI) to investigate the uptake of the particles by cells and 5 random region of interests were evaluated to determine the overall number of PCI, yielding a total of 15 region of interests per treatment.
Randomization	As all samples were prepared identically, randomization is not applicable. During data collection, all region of interests that were imaged were chosen randomly in a sample.
Blinding	The investigators were blinded to treatment group allocation during data evaluation if the evaluation procedures were not fully automated.

Reporting for specific materials, systems and methods

We require information from authors about some types of materials, experimental systems and methods used in many studies. Here, indicate whether each material, system or method listed is relevant to your study. If you are not sure if a list item applies to your research, read the appropriate section before selecting a response.

Materials & experimental systems

n/a	Involved in the study
☒	☐ Antibodies
☐	☒ Eukaryotic cell lines
☒	☐ Palaeontology and archaeology
☒	☐ Animals and other organisms
☒	☐ Clinical data
☒	☐ Dual use research of concern
☒	☐ Plants

Methods

n/a	Involved in the study
☒	☐ ChIP-seq
☒	☐ Flow cytometry
☒	☐ MRI-based neuroimaging

Eukaryotic cell lines

Policy information about cell lines and Sex and Gender in Research

Cell line source(s)	J774A.1: German Collection of Microorganisms and Cell Cultures GmbH (DSMZ). J774A.1 stably transfected with a LifeAct-GFP construct: Gift by Alexander Rohrbach.
Authentication	No further measures were taken to authenticate the cell lines used in this study.
Mycoplasma contamination	All cells used in this study were tested negative for mycoplasma after passage 30, using the PanReac AppliChem PCR Mycoplasma Test Kit (Product-No. A3744).
Commonly misidentified lines (See ICLAC register)	No commonly misidentified cell lines were used in this study.

Plants

Seed stocks	*Report on the source of all seed stocks or other plant material used. If applicable, state the seed stock centre and catalogue number. If plant specimens were collected from the field, describe the collection location, date and sampling procedures.*
Novel plant genotypes	*Describe the methods by which all novel plant genotypes were produced. This includes those generated by transgenic approaches, gene editing, chemical/radiation-based mutagenesis and hybridization. For transgenic lines, describe the transformation method, the number of independent lines analyzed and the generation upon which experiments were performed. For gene-edited lines, describe the editor used, the endogenous sequence targeted for editing, the targeting guide RNA sequence (if applicable) and how the editor was applied.*
Authentication	*Describe any authentication procedures for each seed stock used or novel genotype generated. Describe any experiments used to assess the effect of a mutation and, where applicable, how potential secondary effects (e.g. second site T-DNA insertions, mosiacism, off-target gene editing) were examined.*
